# Triple-Band Perfect Light Absorber Based on Hybrid Metasurface for Sensing Application

**DOI:** 10.1186/s11671-020-03332-x

**Published:** 2020-05-11

**Authors:** Yongzhi Cheng, Fu Chen, Hui Luo

**Affiliations:** School of Information Science and Engineering, Wuhan University of Science and Technology, Wuhan, 430081 People’s Republic of China

**Keywords:** Visible, Hybrid metasurface, Perfect light absorber, Silicon cross nanostructure

## Abstract

A simple design of triple-band perfect light absorber (PLA) based on hybrid metasurface in visible region has been presented in this work, which turns out to be applicable for refractive index (RI) sensing. Distinct from previous designs, the proposed hybrid metasurface for visible PLA is only consisted of periodic silicon cross nanostructure arrays and gold substrate. The periodic silicon cross arrays deposited on the gold substrate contribute to excite the guided modes under the normal incident light illumination. According to the simulation results, it can be found that three perfect absorption peaks of 98.1%, 98.7%, and 99.6% which are located at 402.5 THz, 429.5 THz, and 471.5 THz, respectively, have been clearly observed in PLA. This triple-band perfect absorption effect could be attributed to the intrinsic loss of silicon material originated from the guided mode excitations caused by the standing waves of different orders. It has been confirmed that the perfect absorption properties of the PLA can be easily regulated by changing the geometric parameters of the unit-cell nanostructure. Furthermore, the designed PLA served as a RI sensor can achieve sensitivity of about 25.3, 41.3, and 31.9 THz /refractive index unit (RIU). It can be believed that the proposed design of PLA for RI sensing would provide great potential applications in sensing, detecting, the enhanced visible spectroscopy, etc.

## Introduction

Metasurfaces, as an important branch of optical metamaterials, are two-dimensional (2D) array architectures which are formed by sub-wavelength periodic plasmonic nanostructure consisting of patterned dielectric and metal materials [1, 2]. In recent years, metasurfaces have been extensively investigated since they could be potentially applied in miniaturized free-space optics components, such as lenses, waveplates, and spectral filters, and absorbers [1–10]. One of the outstanding aspects of metasurfaces might be perfect light absorbers (PLAs) operated in optical region since they have promising applications in optical communication [11], thermal emitting [12, 13], light harvesting [14], and sensing [15–17]. Generally, the metasurface-based PLAs could be realized by the configuration of tri-layered metal-dielectric-metal (MDM) nanostructures or bi-layered dielectric-metal (DM) nanostructures, in which the various surface plasmon resonances (SPRs) can be excited and subsequently cause light energy confinement in the patterned metals or metal-dielectric interface [11, 15–28]. In addition, intrinsic optical losses of metallic and dielectric materials in PLA are also the important and advantageous factors for enhancing electromagnetic (EM) energy absorption of the incident lights [11, 19–30]. It has been confirmed that the absorption capacity of PLA is usually depended on the shape, size, thickness, and composition of the plasmonic metasurface, which are also sensitive to the refractive index (RI) of surrounding material [29–36]. As it is well known for sensing applications, the narrowband PLA was investigated intensively due to its large modulation depth [15–18, 31–37]. When the PLA is placed in a gas or liquid environment, the frequency of absorption peak would shift significantly with the change of the RI value of surrounding material. Hence, numerous metasurfaces based on narrowband PLAs have been proposed and investigated intensively [31–38]. For example, Cheng et al. proposed a narrowband PLA based on the MDM configuration, which could achieve sensitivity about 590 nm ∕RIU [31]. Bhattarai et al. demonstrated a mushroom-capped narrowband PLA based on a Fabry-Perot cavity mechanism, and the sensitivity is up to 2508 nm∕ RIU [32]. Then, other PLAs based on MDM configurations have been proposed continuously and investigated theoretically [33–37]. Although these narrowband PLAs could achieve high sensitivity, the large-scale production is time consuming and costly due to their complexity of the metasurface design. Therefore, it would be extremely helpful if narrowband PLA could be supported by relatively simple structures. Yong et al. proposed simple design scheme of the PLAs for the sensing application based on all-metal metasurface [38–40]. For these PLAs, the noble metals gold or silver are usually utilized, which would also increase the fabrication cost.

Recently, the metasurfaces based on silicon nanostructures have attracted great attention due to their applications in detector [41], photonic waveguide [42], color generator and filter [43, 44], and PLAs [45–50]. Similar to the metallic nanostructures, silicon is one of feasible high RI materials which could support various SPRs by structural design at optical frequency range. In addition, silicon also can be cost-effectively and appreciably compatible with complementary metal oxide semiconductor (CMOS) process [44, 49]. Therefore, it can be believed that the narrowband perfect absorption in silicon metasurface-based PLAs would be highly significant in sensing application [50]. For example, Ahmmed et al. proposed a PLA based on hybrid metasurface composed of amorphous silicon nano-disk arrays deposited on a gold layer, which could work as RI sensor in near-infrared region [50]. However, it only works in a single narrowband which restricts potential applications in multiplex sensing detection. To the best of our knowledge, it can be hardly found reports about high efficiency multiple-band PLAs utilizing metasurface, whose operation is valid in visible region.

In this work, a triple-band PLA based on hybrid metasurface in visible region is proposed and demonstrated theoretically, which can be applicable for RI sensing. The hybrid metasurface, consisting of single-sized silicon cross nanostructure arrays on a gold substrate, exhibits a triple-band perfect absorption with absorbance of over 98% at three distinct resonance frequencies. An underlying physical mechanism of the observed perfect absorption has been also illustrated by analyzing spatial distributions of electric fields, power flow, and power loss density at resonances. The impact of the geometric parameters of the unit-cell nanostructure to absorption properties of the PLA has been investigated as well. Furthermore, the absorption peaks of PLA have been confirmed to be sensitive to the RI value of the surrounding medium, thus makes it to be a potential candidate for sensing applications. Besides, the hybrid metasurface-based PLA could be fabricated easily and simply, as well as integrated easily into plasmonic, electronic, and photonic devices on the same chip. Such a design of triple-band PLA paves the effective way to the realization of nano-photonic devices based on hybrid metasurface, which could be a candidate for potential applications in multiplex sensing, detecting, and enhanced visible spectroscopy.

## Methods

**Fig. 1:**
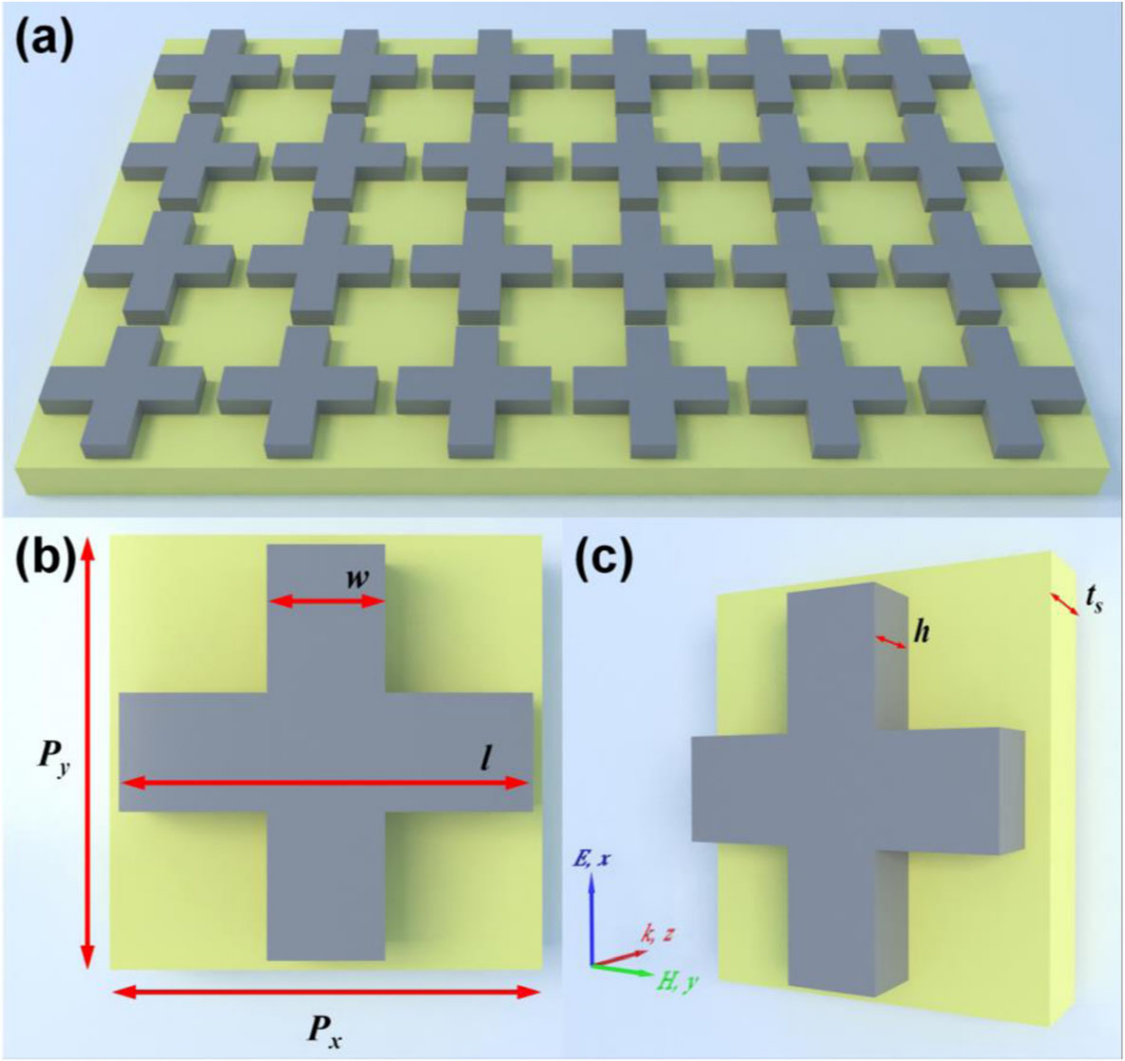
Schematic illustration of visible triple-narrowband PLA. **a** The 2D periodic array structure. **b** Front. **c** Perspective view of the unit-cell nanostructure

Figure 1 presents the design schematic of the visible PLA based on a hybrid metasurface, which only consisted consists of two functional layers: the periodic silicon cross nanostructure arrays constitute the top layer acting as the dielectric resonator, while the bottom layer is the gold substrate. It has been demonstrated that different patterned plasmonic silicon structures can support different SPR modes under incident light illumination, which could be applied to construct the PLAs from terahertz to visible frequency range due to its favorable optical properties [42–48].

In the visible region, the semiconductor silicon is an economical material with high RI which can be regarded as the dielectric resonator by special structural design [43–49]. In addition, there is a salient advantage that the semiconductor silicon can be efficiently grown over a heterogeneous substrate (such as gold substrate) at a low temperature relying on appreciable compatibility with the CMOS process [44, 49], which is easy to meet the requirements of large-scale production. In our interested visible range (350–500 THz), the RI value of the silicon is approximately a constant, which is about *n*_*si*_ = 3.7 × (1 + 0.0025*i*) [50–52]. The gold (Au) substrate layer can be described by the frequency-dependent Drude model from the experiment data [53]. The thickness of the gold substrate is higher than the penetration depth of the incident light in the visible range. Different from the typical MDM configuration, our proposed PLA based on the hybrid metasurface is formed by a sub-wavelength periodic bi-layered DM nanostructure, and it can be expected that the proposed PLA is polarization-independent due to the geometric rotation symmetry of the cross nanostructure and the square-lattice. The optimized geometric parameters of the design are given as follows: *p*_*x*_ = *p*_*y*_ = 400 nm, *l* = 350 nm, *w* = 100 nm, *h* = 85 nm, and *t*_*s*_ = 100 nm. As shown in Fig. 1c, the unit cell of proposed PLA is set to be a constant periodicity of 400 nm along the *x-* and *y*-axis directions to avoid diffraction for frequencies up to 750 THz.

The proposed PLA based on the hybrid metasurface was designed and investigated by means of a simulation tool based on the finite element method (FEM) in CST Microwave Studio. As shown in Fig. 1c, plane wave excitation with a wide frequency range from 350 to 500 THz is considered as the illumination source with a wave vector which is normal to the surface of hybrid metasurface. In simulation, the mesh size is set as 0.3 nm, which is much smaller than the operating wavelength and the unit-cell size. To ensure negligible numerical errors, we also performed the standard convergence test before simulation of the unit cell. The periodic boundary conditions along *x-* and *y*-axis directions are used to consider periodic arrangement of the hybrid metasurface. The incident linear polarization light is set to be propagating along the z-axis direction in a manner that the electric (*E*_*x*_) and magnetic (*H*_*y*_) fields are along the *x*- and *y*-axis directions, respectively. In our design, since the transmission is blocked off by the gold substrate, the absorbance could be calculated only by *A*(ω) = 1 - *R*(ω) = |*S*_11_|^2^, where *S*_11_ is the reflection coefficient.

## Results and Discussions

**Fig. 2:**
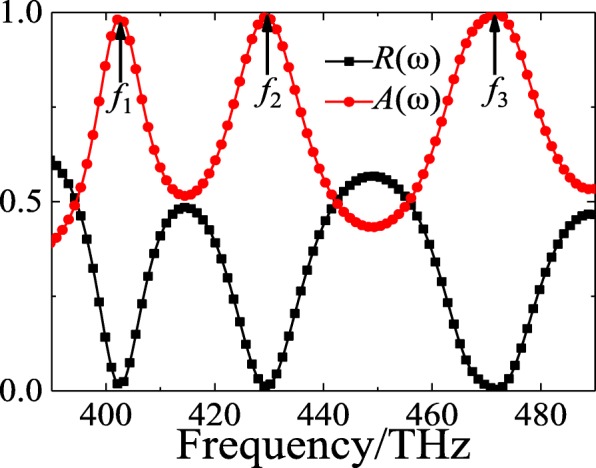
The simulated reflectance (*R*(ω)) and absorbance (*A*(ω)) spectra of the designed visible hybrid metasurface-based PLA under normal incident light illumination

Figure 2 presents the simulated reflectance and absorbance spectra of the PLA based on hybrid metasurface under normal incident light illumination in visible region. Three distinct resonance points are evidently observed at *f*_1_ = 402.5 THz, *f*_2_ = 429.5 THz, and *f*_3_ = 471.5 THz, respectively. At these resonances, the reflectance is decreased to 1.9%, 1.3%, and 0.4%, and the corresponding absorbance increase to the maximal values of 98.1%, 98.7%, and 99.6%, respectively. According to previous works [45–48], it could be conjectured that the perfect absorption at three resonances could be attributed to the excitations of higher-order SPR modes in silicon cross nanostructures under normal incident lights illumination which will be discussed later. Although both the high RI semiconductor silicon and high reflectance gold substrate which are widely used in previous works have been applied in our design [17, 38–40, 46, 48–50], it is still worth to be pointed out that the proposed novel design of PLA in this work exhibits a relatively improved property which is in terms of a triple-band perfect absorption in visible region by utilizing single-sized silicon cross nanostructure. In addition, it can be expected that the proposed PLA should be polarization-insensitive for the normal incident light due to its high geometric rotation symmetry of the unit cell which is similar with the previous designs [54–56].

In addition, the full width at half maximum (FWHM) and Q-factor of the proposed PLA have been also calculated according to the previous reference [40]. At those three resonance positions mentioned above, the value of FWHM is about 64.875 THz, 27.75 THz, and 34.125 THz, and the corresponding Q-factor (=*f*_i_/FWHM_i_, *i* = 1, 2, 3) is about 6.48, 14.57, and 13.82, respectively. It should be noticed that the triple-band perfect absorption can be observed in the ideal case with air media. However, it is possible to tune the resonance absorption property by adjusting the outside/environment RI value of the designed PLA. It means that the operation frequency could be significantly regulated by changing the RI value of the environment surrounding on the PLA. Thus, the designed PLA with steep resonances can provide some potential applications in multiplex sensors and detectors.

**Fig. 3:**
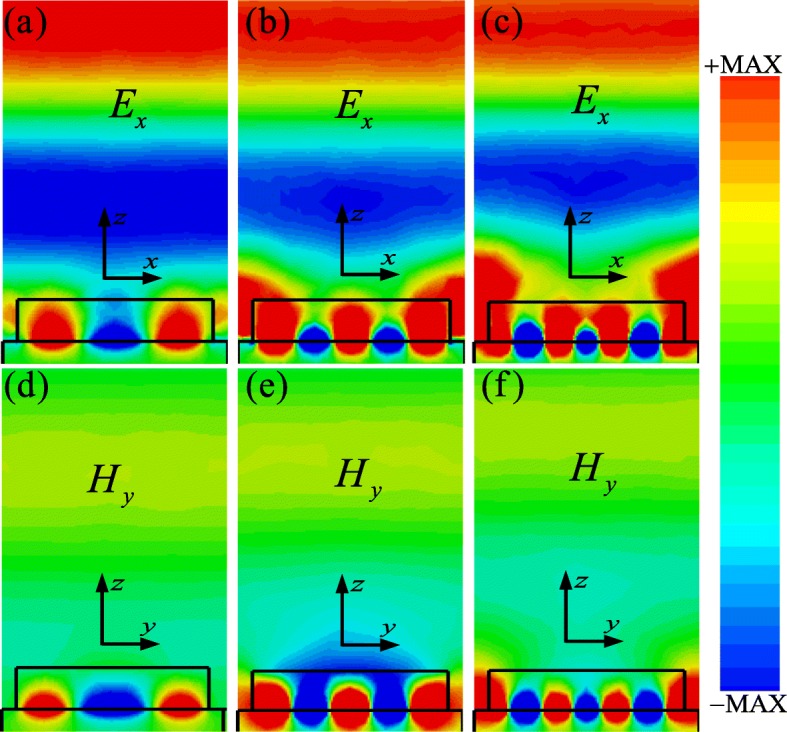
Distributions of the **a**–**c** electric field (*E*_*x*_ in the *x-z* plane of *y* = 0 nm) and **d**–**f** magnetic field (*H*_*y*_ in the *y-z* plane of *x* = 0 nm) in the unit-cell nanostructure of the PLA at different resonance frequencies: (**a**, **d**) *f*_1_ = 402.5 THz, (**b**, **e**) *f*_2_ = 429.5 THz, and (**c**, **f**) *f*_3_ = 471.5 THz

To verify the physics mechanism behind the observed triple-band perfect absorption phenomenon of the designed PLA, the spatial distributions of electric (*E*_*x*_, *x-z* plane) and magnetic (*H*_*y*_, *y-z* plane) field at those three absorption peaks have been systemically investigated, as shown in Fig. 3. Obviously, the spatial distribution patterns of the strong electric and magnetic fields (*E*_*x*_ and *H*_*y*_) are significantly different at various resonance frequencies, revealing the excitations of different SPR modes. However, it is evident that both electric and magnetic field are always strongly concentrated in the interface of the silicon cross and gold substrate when the resonance occurs. These spatial field features indicate that the guided modes with different higher orders in the interface of the silicon cross nanostructure and gold substrate have been excited. It can be believed that the intense guided mode resonances at the dielectric/metal interface are excited when incident light is coupled between waveguides with different refractive indices [57–60]. Meanwhile, the resonant couplings between the incident light and the guided mode of the dielectric/metal nanostructure are possible, which is similarly to the metal grating guided mode resonance effect [21, 59, 60].

To illustrate the guided mode resonance of the designed PLA nanostructure, we can assume the designed silicon cross nanostructure as a dielectric waveguide in visible region. When the incident light impinges the gaps between the adjacent two unit cells, it would be diffracted into the silicon layer and then reflected by the gold substrate, subsequently guided into the interface of the silicon/gold substrate. Due to the symmetrical design of the unit cell, the coupling guided lights from adjacent gaps propagate oppositely and consequently combine to form a standing wave in the waveguide layer [58–60]. According to these results shown in the Fig. 3a–f, it can be found that only odd harmonic guided modes in the nanostructure can be excited under the normal incident light illumination. Figure 3a–f shows the first-order mode, third-order mode, and fifth-order mode in the nanostructure, respectively. The results are well consistent with the previous PLAs based on MDM configuration [58, 61], in which the second-order mode could not be excited for the normal incident lights. It is because that the excitations of the harmonic guided modes are mainly determined by the geometric parameters of the designed nanostructure. It means that only odd or harmonic-guided modes could be excited under the special appropriate nanostructure design in this work. The guided-mode excitations with higher orders in this nanostructure would contribute to enhance the incident lights coupling into the air gap and localizing in the silicon/gold interface, finally creating a perfect light absorption at various resonance frequencies. As it is well known, the energy loss of incident light induced by the guided modes excitation in the nanostructure is always large enough to introduce the high level absorption at resonances [20, 21, 26, 58–61]. Besides, these guided mode resonances are mainly determined by geometric sizes and surrounding media of the designed nanostructure [58]. It can be concluded that the higher-order-guided modes could be also applied to obtain the high-performance PLA in the visible region with moderate geometric parameters in comparison to using the fundamental mode with deeper sub-wavelength structure [61].

**Fig. 4:**
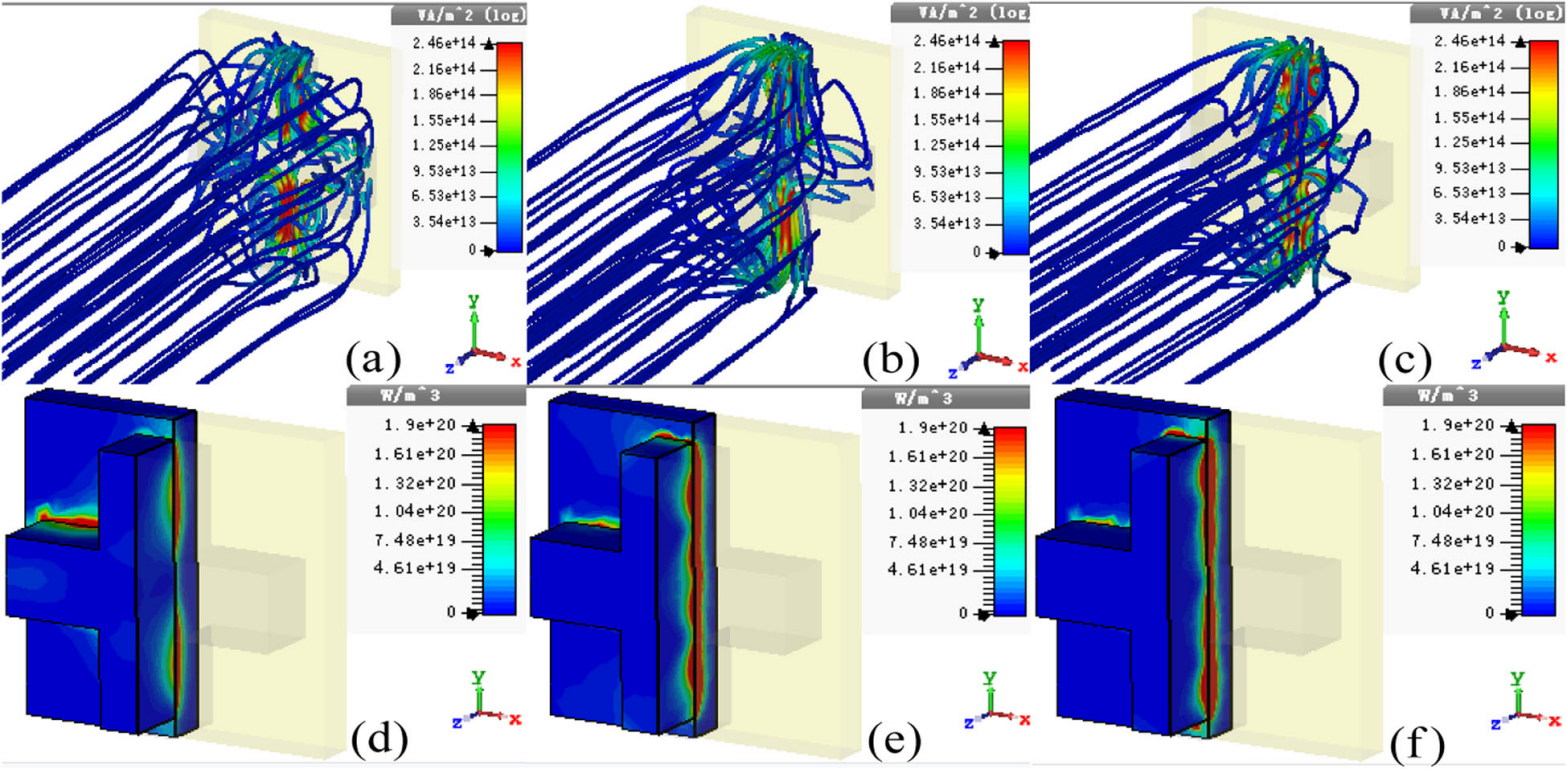
The three-dimensional (3D) distributions of the **a**–**c** power flow stream and **d**–**f** power loss density of the PLA at different resonance frequencies: (**a**, **d**) *f*_1_ = 402.5 THz, (**b**, **e**) *f*_2_ = 429.5 THz, and (**c**, **f**) *f*_3_ = 471.5 THz

To get a deeper and qualitative understanding of the perfect absorption above, the 3D distributions of power flow stream and power loss density for normal incident *y*-polarized lights at various resonance frequencies (*f*_1_ = 402.5 THz, *f*_2_ = 429.5 THz, and *f*_3_ = 471.5 THz) have been also studied, as illustrated in Fig. 4a–c. Firstly, the input light power flows are originally parallel streams in the space far away from the nanostructure at resonances. When incident light flows get closer to the PLA, most of them flow “across” the unit cell, subsequently curl in the silicon layer and finally concentrate on the interface of the silicon and gold substrate. In this case, the spatial form of the power flower streams in nanostructure exhibits various characteristics at different absorption frequencies. The power flow stream profiles caused by the guided-mode excitations take place in the nanostructure, and the intrinsic loss usually happens in bulk materials. Owing to the dielectric loss nature of silicon and gold in the visible region, it can be considered that the light energy losses induced by the guided-mode excitations with different higher orders should be mainly originated from the silicon cross nanostructure and gold substrate.

Figure 4d–f illustrates the 3D distributions of power loss density in the unit-cell nanostructure at frequencies of *f*_1_ = 402.5 THz, *f*_2_ = 429.5 THz, and *f*_3_ = 471.5 THz, respectively. It can be observed that the power loss densities mainly distribute in the interface between the silicon cross nanostructure and gold substrate. Obviously, the power of incident light is completely confined in the designed PLA nanostructure. Since the silicon and gold in the nanostructure are both dielectric loss material in visible region, thus the light energy dissipation takes place in the designed PLA [48, 49]. In our design, the silicon cross is much more favorable for improving the absorption performance than the previous square and disk, since the gaps of the proposed cross type nanostructure would easily capture more incident lights due to the guided mode excitations [47–49]. As a matter of fact, the structured silicon with appropriate geometrical design itself can serve as a good PLA, relying on the lossy feature of silicon material in visible region [49]. Furthermore, the silicon cross could be also assumed as an anti-reflection layer, which makes the gold substrate to be a nearly perfect absorption material at resonances. The gold is still plasmonic in the visible region since the real part of its permittivity is negative [53]. It should be noticed that the incident light will be strongly repulsed by gold substrate, and the perfect absorption would be impossible without SPRs response.

Relying on the analyses above, it could be concluded that the triple-band perfect absorption of proposed PLA is originated from the guided modes with higher-order and dielectric loss nature of silicon and gold substrate in the visible region. In a word, the guided-mode resonance and losses of the nanostructure are the two key factors for the perfect absorption of the designed PLA.

**Fig. 5:**
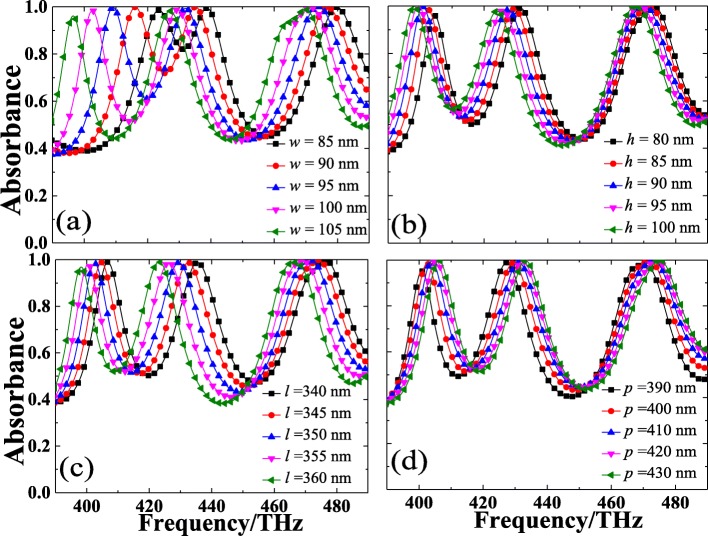
The dependence of the perfect absorption on different geometric parameter of the proposed PLA. **a**–**c** Wire width (*w*), height (*h*), wire length (*l*) of the silicon cross nanostructure, and **d** periodicity (*p*) of the unit cell

Next, the influences of geometric parameters for each unit cell on the absorption property of our design PLA have been systematically investigated by a parametric study. As for the proposed PLA in this work, only four geometric parameters are needed to be considered: wire width (*w*), wire length (*l*), height (*h*) of silicon cross nanostructure, and the periodicity (*p*) of the unit cell. A series of absorbance spectra of the designed PLA with different geometric parameters (*w*, *h*, *l*, and *p*) have been illustrated in Fig. 5a–d. It is worthy to be noticed that only one geometric parameter could be regulated at a time, while the others remain constant.

On the basis of Fig. 5a, b, it can be observed that the absorbance of resonance peaks can be maintained over 95% when changing the one geometric parameter while others remain constant. However, the operation frequency is found to be sensitive to the geometric parameters of the PLA. When the periodicity (*p*) of the PLA is fixed, the absorption peak frequency turns out to be inversely proportional to the geometric sizes (*w*, *h*, and *l*) of the unit cell, which is in well consistent with previous studies [58, 62]. This is because the effective refractive index of the guided-mode resonances increases with the augment of *w*, *h*, and *l*. The absorbance properties of the PLA with different sizes of *w* have been depicted in Fig. 5a. With changing *w* from 85 to 105 nm by step of 5 nm, distinguished red-shift of absorption spectral can be observed clearly. For the PLA with a wider wire width (*w* > 100 nm) of silicon cross, the absorbance of the first and second absorption peaks will decrease slightly, but the third one can be nearly maintained. This kind of response mainly results from the weakened coupling and confinement effect induced by the nanostructure. In addition, compared with the second and third resonance peaks, it can be found that the first peak is much more sensitive to the variations of the wire width *w*, resulting in a salient red-shift phenomenon. The absorbance properties of the PLA with different sizes of *h* have been presented in Fig. 5b. When the height *h* increases from 80 to 100 nm in intervals of 5 nm, the absorption spectra variations are similar to the case of changing the wire width *w*, and the absorption peak frequencies exhibit a slight red-shift as well. With the increasing of *h*, it can be found that the absorbance of the first resonance peak gradually increase while the second one decrease slightly, and the third one can be nearly maintained at constant. As shown in Fig. 5c, it can be found that the absorption peaks will shift to the lower frequencies when the wire length *l* increases from 340 nm to 360 nm by a step of 5 nm. As the increasing of wire length *l*, the absorbance of the first absorption peak decreases slightly while the other resonance peaks remain constant. As shown in Fig. 5d, a completely contrary variation tendency, which can be described in terms of a “blue-shift” of the absorption peaks, has been found when the periodicity *p* increase from 390 to 430 nm in intervals of 10 nm. With the increasing of periodicity *p*, the absorbance of the first resonance peak increases slightly while the other absorption peaks are nearly unchanged. In sum, the results illustrated in Fig. 5 confirm that these absorption peaks are related with characteristics of standing waves which have been demonstrated in Fig. 3, indicating that the operation frequency and efficiency of the proposed PLA could be directly regulated by the relative geometric parameters including wire width (*w*), height (*h*), wire length (*l*), and periodicity (*p*).

**Fig. 6:**
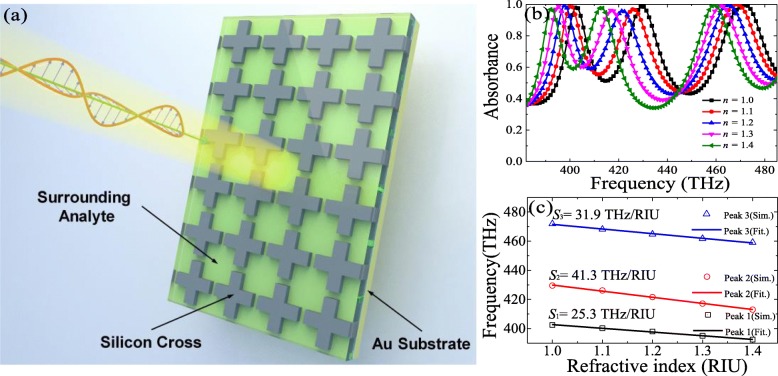
**a** The schematic of the PLA for RI sensing application. **b** the simulated absorbance spectra of the PLA by varying the RI values of surrounding analyte from *n* = 1.0 to *n* = 1.4 by step of 0.1. **c** Linear fit (solid lines) and simulated resonance frequencies (hollow symbols) as a function of RI values of the surrounding analyte

According to the results and discussions of the designed triple-band PLA above, it could be expected as a promising candidate for RI sensing application. To clarify the practicability of our designed triple-band PLA for sensing applications, the behavior of absorbance spectra as a function of RI values of the surrounding analyte has been further verified. As shown in Fig. 6a, the surrounding analyte is filled in gaps of the silicon cross nanostructure of the proposed PLA. Since our PLA has a triple narrow bandwidth and perfect absorption around the resonant frequency, it could be expected to exhibit a good sensing performance. The dependence of the absorbance spectra on the change of the RI value of the surrounding analyte has been presented in Fig. 6b. It should be noticed that the absorbance could be maintained over 95% when the RI value of the surrounding analyte changes from *n* = 1.0 to *n* = 1.4 with a step of 0.1, while frequency shifts of the three resonance peaks are quite conspicuous which could be described in terms of an obvious red-shift with the increasing RI value of the surrounding analyte. The variations of the frequency points 1 (*f*_1_), 2 (*f*_2_), and 3 (*f*_3_) turn out to be about 2.53 THz, 4.13 THz and 3.19 THz on average, respectively. In fact, the sensing capability of the PLA has been widely accepted to be described by a definition of bulk RI sensitivity (S): *S* = Δ*f*/Δ*n*, where Δ*f* and Δ*n* are the change of the resonance frequency and RI value, respectively [63]. According to the definition above, as shown in Fig. 6c, the average *S* values of three frequency points (*f*_1_, *f*_2_, and *f*_3_) are evaluated to be about 25.3, 41.3, and 31.9 THz/RIU, respectively. Owing to the excellent sensing characteristic, the design of the triple-band PLA could be believed to be promising in sensor related fields.

## Conclusions

In conclusion, a simple design of the triple-band PLA based on hybrid metasurface has been proposed and investigated numerically in this work, which can be believed to be applicable for RI sensing. The proposed PLA based on the hybrid metasurface is designed to be only consisted of periodic arrays of silicon cross nanostructures deposited on a gold substrate. The numerical results indicate that the designed PLA can exhibit a relatively high absorbance of 98.1%, 98.7%, and 99.6 % at 402.5 THz, 429.5 THz and 471.5 THz, respectively. The physical pictures of the designed PLA have been explored by analyzing the spatial distributions of the electric and magnetic field at three different resonance frequencies. It turns out to be that the EM energy could be dissipated through the standing waves originated from different higher-order-guided modes in the lossy interface between silicon cross nanostructure and gold substrate, leading to the triple-band perfect absorption. Besides, the spatial distributions of the power flow stream and loss density reveal that the dielectric loss feature of silicon and gold in the visible region is also critical for the perfect absorption of the PLA. In addition, the resonance absorption properties of our designed PLA nanostructure have been also confirmed to be well tuned in the visible region by regulating the geometric parameters of unit cell. Furthermore, the frequencies of the resonance peaks have been demonstrated to be very sensitive to the RI variations of the surrounding analyte filled in the proposed PLA. The average bulk RI sensitivity *S* values of the PLA are about 25.3, 41.3, and 31.9 THz/RIU, respectively. The proposed PPA is easy to fabricate by the deep reactive ion etching (DRIE) or advanced electron beam lithography (EBL) technique, which is cost-effectively compatible with CMOS process [44, 49]. Therefore, this design of the PLA can open a new avenue for multispectral RI sensing applications in the visible region, especially for bio-molecular, gas detection, medical diagnostics, and spatial biosensing. It also has potential in applications of substrates for multiplex sensing activities of differentiation and proliferation of neural stem cells.

## Supplementary information

**Additional file 1.**

## Data Availability

The datasets generated and/or analyzed during the current study are available from the corresponding author on reasonable request.

## Abbreviations

PLA: Perfect Light Absorber
RI: Refractive index
RIU: Refractive index unit
2D: Two-dimensional
MDM: Metal-dielectric-metal
SPRs: Surface plasmon resonances
EM: Electromagnetic
CMOS: Complementary metal oxide semiconductor
FEM: Finite element method
FWHM: Full width at half maximum
DRIE: Deep reactive ion etching
EBL: Electron beam lithography
