# Supplementary figures and images for: Triple-Band Perfect Light Absorber Based on Hybrid Metasurface for Sensing Application
Source: Nanoscale Res Lett. 2020 May 11;15:103. doi: 10.1186/s11671-020-03332-x (PMC7214565; doi:10.1186/s11671-020-03332-x)

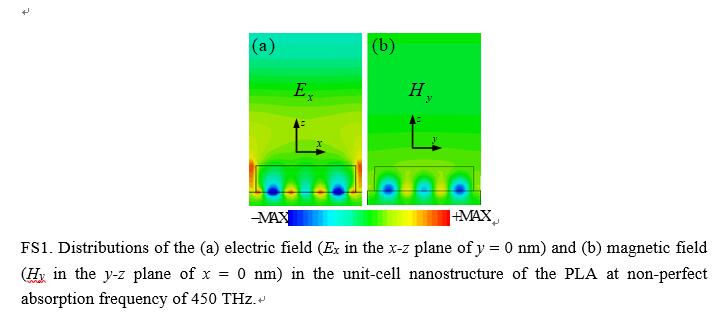

Supplement: Supplementary file 1 — Additional file 1. [file 11671_2020_3332_MOESM1_ESM.jpg]
